
JOURNAL INFORMATION
==============================
NLM Title Abbreviation: Exp Physiol
Journal ID: Exp Physiol
NLM Journal ID: 9002940
Journal ID: EPH

Experimental Physiology

ISSN: 0958-0670
EISSN: 1469-445X
Publisher: Wiley

ARTICLE INFORMATION
==============================
PMCID: PMC13131075
DOI: 10.1113/eph.70289
Article ID: EPH70289
Article version: 1
Subjects: Issue Information, Issue Information

Issue Information

Electronic publication date: 2026 Apr 30
Publication date: 2026 May
Volume: 111
Issue: 5
First page: 2731
Last page: 2735
Copyright: © 2026 The Authors. Experimental Physiology published by John Wiley & Sons Ltd on behalf of The Physiological Society.
License: This is an open access article under the terms of the http://creativecommons.org/licenses/by/4.0/ License, which permits use, distribution and reproduction in any medium, provided the original work is properly cited.
License URL: https://creativecommons.org/licenses/by/4.0/

Figure count: 0
Table count: 0
Page count: 5

source-schema-version-number: 2.0
cover-date: 1 May 2026
details-of-publishers-convertor: Converter:WILEY_ML3GV2_TO_JATSPMC version:6.7.6 mode:remove_FC converted:30.04.2026

==============================

### Experimental Physiology

Open Access

Integration and Translation

Volume 111 | Issue 5 | 1 May 2026

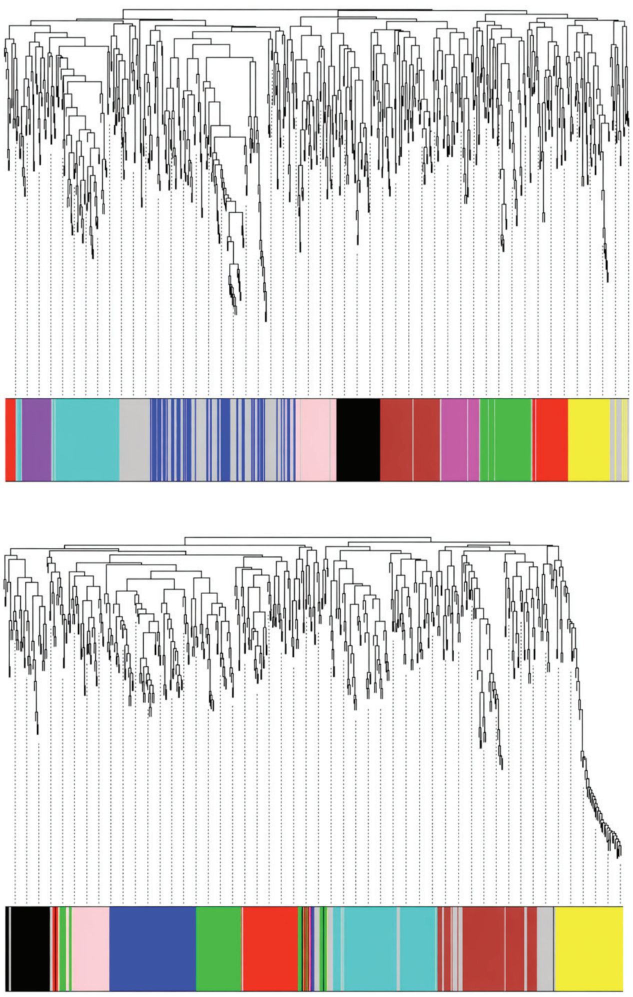

WILEY

The Physiological Society

150 YEARS 1876 2026 LEADING DISCOVERY SCIENCE

Experimental Physiology

Open Access

Integration and Translation

Volume 111/Issue 5/
1 May 2026

### EDITORIAL BOARD

**EDITOR-IN-CHIEF**
Damian Bailey *Pontypridd, UK*

**DEPUTY EDITOR-IN-CHIEF EUROPE**
Ronan Berg *Copenhagen, Denmark*

**DEPUTY EDITOR-IN-CHIEF USA**
David Poole *Manhattan, KS, USA*

**SENIOR ETHICS EDITOR**
Susan Barman *East Lansing, MI, USA*

**STATISTICAL EDITOR**
Cody Durrer *Copenhagen, Denmark*

**SENIOR REVIEWS EDITOR**
Toby Mündel *Ontario, Canada*

**CONNECTIONS EDITOR**
Prem Kumar *Birmingham, UK*

**SENIOR EDITORS**
Dana Almohazey *Dammam, Saudi Arabia*
Jill Barnes *Madison, WI, USA*
R. Matthew Brothers *Arlington, TX, USA*
Kanokwan 'Mandy' Bunsawat *Iowa City, IA, USA*
Kirstene Callø *Copenhagen, Denmark*
Katrina Colafella *Victoria, Australia*
Ellen Dawson *Liverpool, UK*
Colleen Deane *Southampton, UK*
Peter Zu Eulenburg *Munich, Germany*
Federico Formenti *London, UK*
Julien Ochala *Copenhagen, Denmark*
Shigehiko Ogoh *Kawagoe, Japan*
Jason Peart *Gold Coast, Australia*
Peter Rasmussen *Copenhagen, Denmark*
Lee Romer *London, UK*
Harry B. Rossiter *Los Angeles, CA, USA*
Bill Sheel *Vancouver, Canada*
Mike Stembridge *Cardiff, UK*

**REVIEWING EDITORS**
Tatsuro Amano *Niigata, Japan*
Claire Badenhorst *Palmerston North, NZ*
Tracy Baynard *Boston, MA, USA*
Hannah Caldwell *Copenhagen, Denmark*
Havovi Chichger *Cambridge, UK*
Silvia Conde *Lisbon, Portugal*
Joseph Costello *Portsmouth, UK*
Jennifer Davies *Cardiff, UK*
Judy Delp *Tallahassee, FL, USA*
Aimee Drane *Cardiff, UK*
Esther Dupont-Versteegden *Lexington, KY, USA*
Andrea Fellet *Buenos Aires, Argentina*
Carrie Ferguson *Torrance, CA, USA*
Igor Fernandes *Niteroi, Brazil*
Ryan Hoiland *Kelowna, Canada*
Seth Holwerda *Kansas City, KS, USA*
Anna Hudson *Adelaide, Australia*
Matthew Kay *Washington, DC, USA*
James Lang *Ames, Iowa, USA*
Jacqueline Limberg *Columbia, MO, USA*
Fiona McBryde *Auckland, NZ*
Carmen De Miguel *Birmingham, AL, USA*
Niels Olesen *Copenhagen, Denmark*
Leah Reznikov *Gainesville, FL, USA*
Kleiton Silva *Camden, NJ, USA*
Sarah Short *Iowa City, IA, USA*
Nathaniel Szewczyk *Nottingham, UK*
Can Ozan Tan *Boston, MA, USA*
Mike Tymko *Vancouver, Canada*
Kai Yang *Guangzhou, China*
Song Yao *Melbourne, Australia*
Gensheng Zhang *Hangzhou, China*

**FUTURE LEADERS**
Edward Ashworth *Basel, Switzerland*
Sophie Broome *Melbourne, Australia*
Callum Brownstein *Newcastle, UK*
Daniel Craighead *Minneapolis, MN, USA*
Robert Lakin *Toronto, Canada*
Jedd Pratt *Manchester, UK*
Fabio Sarto *Padua, Italy*
Hollie Speer *Canberra, Australia*

**CONSULTANT EDITORS**
Richard Burden *Bisham, UK*
Nic Green *Bedfordshire, UK*
Julie Greeves *Salisbury, UK*
Denis Noble *Oxford, UK*
Nanduri Prabhakar *Chicago, IL, USA*

**PUBLISHER**
Alex Stewart (astewart@physoc.org)

**DEPUTY MANAGING EDITOR**
Hollie Evans (hevans@physoc.org)

**PEER REVIEW MANAGER**
Diana Jones (ephjournal@physoc.org)

**PEER REVIEW COORDINATOR**
Paroma Guha (ephjournal@physoc.org)

**EDITORIAL OFFICE**
The Physiological Society
Hodgkin Huxley House
30 Farringdon Lane, Clerkenwell
London EC1R 3AW

**PRODUCTION OFFICE**
K. Nandini (EPH@wiley.com)

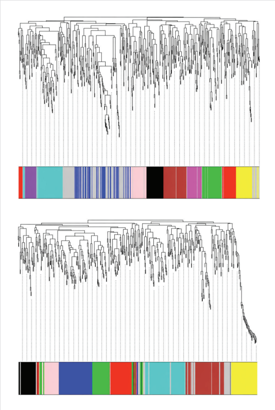

**Cover Illustration**
Clustering dendrograms of metabolites co-expressing in module assignments for serum (top) and skeletal muscle (bottom). Coloured bars indicate module assignments for clustered metabolites. See Drummer et al. Pages 2613–2626.

**Open Access and Copyright**
All articles published by Experimental Physiology [submitted after 18 October 2022] are fully open access: immediately freely available to read, download, and share. All articles [accepted from 18 October 2022] are published under the terms of a Creative Commons license.
Copyright on any research article published by Experimental Physiology is retained by the author(s).
Further information about open access licenses and copyright can be found at: https://authorservices.wiley.com/author-resources/Journal-Authors/licensing/licensing-info-faqs.html#4. Information on the licenses used by Experimental Physiology can be found at: https://physoc.onlinelibrary.wiley.com/journal/1469445X.

For submission instructions and all other information visit: https://physoc.onlinelibrary.wiley.com/journal/1469445X

The Physiological Society and The Physiological Society logo are trade marks belonging to The Physiological Society and are registered in the UK and in the EU Community, respectively.

*Experimental Physiology* publishes high quality, original, physiological research papers that give novel insights into fundamental homeostatic and adaptive responses in health and papers that further our knowledge of pathophysiological mechanisms in diseases.

Experimental Physiology
Open Access
Integration and Translation

### INFORMATION AND GUIDANCE FOR AUTHORS

### Full version online at http://ep.msubmit.net/cgi-bin/main.plex?form_type=display_requirements

### ORIGIN

*The Quarterly Journal of Experimental Physiology*, founded in 1908 by Sir Edward Sharpey-Schafer, Professor of Physiology, University of Edinburgh, became a publication of The Physiological Society in 1981. The title was changed to *Experimental Physiology* in 1990.

### SCOPE

*Experimental Physiology* publishes research papers that report novel insights into homeostatic and adaptive responses in health, as well as those that further our understanding of pathophysiological mechanisms in disease. We encourage papers that embrace the journal's orientation of translation and integration, including studies of the adaptive responses to exercise, acute and chronic environmental stressors, growth and aging, and diseases where integrative homeostatic mechanisms play a key role in the response to and evolution of the disease process. Examples of such diseases include hypertension, heart failure, hypoxic lung disease, endocrine and neurological disorders. We are also keen to publish research that has a translational aspect or clinical application. Comparative physiology work that can be applied to aid the understanding human physiology is also encouraged.

Manuscripts that report the use of bioinformatic, genomic, molecular, proteomic and cellular techniques to provide novel insights into integrative physiological and pathophysiological mechanisms are welcomed.

We are also interested in methodological papers reporting important new developments in physiological techniques that could lead to significant new insights.

Papers which observe a significant new physiological behaviour or phenomenon which has the potential to alter the understanding of that field, but which have not yet identified the mechanisms may also be considered. Such papers should demonstrate the experimental examination and exclusion of a number of plausible mechanisms for the important novel phenomenon.

**Article Types**

In addition to original research, *Experimental Physiology* consider the following article types for publication:

*Case studies, Short Communication, Connections, Reviews, Full Reviews, Hot Topic Reviews Myths and Methodologies and Symposium Reports.*

Review articles are normally by invitation but proposals may be submitted for consideration.

Commentary articles in the form of Viewpoints and Letters are also published.

For full guidelines on all published article see full guidelines.

C O P E COMMITTEE ON PUBLICATION ETHICS

This journal is a member of and subscribes to the principles of the Committee on Publication Ethics

### ETHICS OF EXPERIMENTATION

**Animal Experiments** - see full journal policy at https://physoc.onlinelibrary.wiley.com/hub/animal-experiments

Experimental Physiology endorses the ARRIVE Guidelines for reporting *in vivo* experiments: http://onlinelibrary.wiley.com/doi/10.1113/expphysiol.2010.053793/full

Authors of papers submitted to Experimental Physiology must adhere to the journals' principles and standards for reporting animal experiments.

Authors should read these guidelines to ensure that their research and experiments adhere to our exacting standards.

Euthanasia/humane killing: Animals must be killed using methods approved for that species, stage of development and size. The standards required by the journals are those of the UK/European Union legislation, set out in in ASPA Schedule 1 in the UK and in Annex IV in the European Directive 2010/63/EU. Methods not on Schedule 1 or Annex IV could be permissible but even where the method used conforms with national guidelines, the onus is on the author to provide scientific evidence to demonstrate that it is at least as humane as those on Schedule 1 and Annex IV.

Our guidelines have been outlined in the Editorial 'Principles and standards for reporting animal experiments in Experimental Physiology'.

**Confirmation of compliance**

Authors must state in the Methods section that the investigators understand the ethical principles under which the journal operates and that their work complies with the animal ethics checklist as outlined in the Editorial.

**Human Experiments** - See full journal policy at https://physoc.onlinelibrary.wiley.com/hub/human-experiments

The Editorial Boards are concerned that all work published in The Journal of Physiology and Experimental Physiology should have been conducted according to the highest standards of safety and ethics. Legislation and accepted practice concerning human experimentation vary from country to country and consequently it is difficult to define absolute requirements. However, work with human subjects should conform to the standards set by the Declaration of Helsinki (last modified in 2013), the Medical Research Council's online guidance on the use of human tissue, and the guidelines set out below. The objectives must be to ensure that all risks are minimized and that subjects are not injured and do not feel they have been abused as a result of participating in the study. Any definition of abuse will include excessive or unexpected pain or discomfort experienced during the experiment. Note, in the case of experiments involving minors, any risks must have been considered absent or minimal, and evidence must be presented that the experiments were performed with the understanding and consent of the legal guardian.

All manuscripts must contain statements indicating that (i) informed consent has been obtained, preferably in writing, (ii) studies conformed to the standards set by the Declaration of Helsinki or the version that was in place at the time of the experiments, NOTE: If the research study was registered (clause 35 of the Declaration of Helsinki) the registration database should be indicated, else the lack of registration should be noted as an exception (e.g. The study conformed to the standards set by the Declaration of Helsinki, except for registration in a database.); and (iii) the procedures have been approved by the local ethics committee.

### DISCLAIMER

The Publisher, the Physiological Society and Editors cannot be held responsible for any errors in or any consequences arising from the use of information contained in this journal. The views and opinions expressed do not necessarily reflect those of the Publisher, the Physiological Society or Editors, neither does the publication of advertisements constitute any endorsement by the Publisher, the Physiological Society, Editors, or Authors of the products advertised.

Wiley Open Access articles posted to repositories or websites are without warranty from Wiley of any kind, either express or implied, including, but not limited to, warranties of merchantability, fitness for a particular purpose, or non-infringement. To the fullest extent permitted by law Wiley disclaims all liability for any loss or damage arising out of, or in connection with, the use of or inability to use the content.

### Experimental Physiology

Vol 111.5

### Contents of volume 111.5

1 May 2026

### EDITORIAL

2397 The physiology of survival: Heat
*Josh Foster, Robert D. Meade and Tom Matthews*

2403 The physiology of survival: Breath-hold shallow-water diving
*Andrew H. Baker, Ashley Jones, Adrian Mayhew, Carlene McAvoy, Ross Macleod, Hugh Montgomery, Craig Robertson, Jo Talbot and Mike Tipton*

2411 The Physiology of Lived Experience: And then there was one – the story of a radical nephrectomy
*Michael J. Shattock*

2416 Against erasure: Restoring Dr Florence Buchanan's missing image in physiology
*Damian M. Bailey*

2423 On the mechanism of control of the pulse rate
*Mark I. M. Noble and Angela J. Drake-Holland*

### CONNECTIONS

2426 Cerebral blood flow response to dynamic resistance exercise
*Stephanie Korad, Toby Mündel and Blake G. Perry*

### SHORT COMMUNICATION

2430 Intra-set rest preserves barbell velocity during a free-weight back squat exercise by shifting energy system contribution
*Erik Hobein, Antonia Edel, Ivan Jukic, Alexander Ferrauti and Thimo Wiewelhove*

### REVIEW

2438 The principles of systems biology
*Denis Noble and Reine Bourret*

2449 Psychobiotics and the microbiota–gut–brain axis: Emerging paradigms in mental health modulation
*Amir Arsalan Ghahari, Mehrdad Nourizadeh, Mehrdad SalekShahabi, Shaghayegh Davari and Saeid Mohammadzadeh Mounesyar*

2464 Understanding stressors in combination: a continued challenge for human performance
*Katrina Hinde, Jennifer Wright and Ella Walker*

2476 Exercise-induced dynamic hyperinflation in chronic obstructive pulmonary disease
*Rebecca F. D'Cruz, Dominic Wilkins and Caroline J. Jolley*

2485 Oxygen diffusion kinetics, buffering capacity and phenotype: A narrative review of oxygen physiology and later phenotype in very preterm infants
*Chad C Andersen, Danielle N Bailey, Tara M Crawford and Michael J Stark*

### RESEARCH ARTICLE

### CARDIOVASCULAR CONTROL

2494 Acute cardiovascular responses to slow and deep breathing in normotensive men and women
*Malika Felton, Vikram Mohan and Vanora A. Hundley*

2518 Paternal sleep deprivation and cardiac dysfunction in offspring: A perspective of myocardial hypertrophy
*Yingying Zhang, Leyao Wang, Jie Wang, Yun Pan, Wen Sun, Zhiwei Zou, Wenlin Bai, Ruiling Fang, Yue Zhang, Ping Zhang, Yuanbao Zhang, Lijian Lei and Wenping Zhang*

### ENVIRONMENTAL AND EXERCISE

2528 Harmonic and power spectrum analysis of vertical ground reaction force in patients who have a stroke within 3 months
*Li Zhang, Hongfang Yao, Shuisheng Fu and Jung Hung Chien*

2543 Estimated exercise-induced maximal myocardial blood flow in untrained and endurance-trained men
*Ilkka Heinonen, Kari K. Kalliokoski, Juhani Knuuti and Marko S. Laaksonen*

2553 Sex and age differences in mechanical, thermal and wetness perception across skin sites at risk of pressure ulcers: A quantitative sensory testing study
*Miriam Martini, Arianna Ortis, Davide Filingeri, Peter R. Worsley, Evelin Makuc, Manuela Deodato, Luigi Murena, Paolo Manganotti and Alex Buoite Stella*

2564 Skeletal muscle mitochondrial marker responses to a single bout and 6 weeks of high load versus high volume resistance training in previously trained men
*Breanna Mueller, Carlton D. Fox, Hailey A. Parry, Paulo H. C. Mesquita, Christopher G. Vann, Bradley A. Ruple, Casey L. Sexton, Joshua S. Godwin, Mason M. McIntosh, Darren T. Beck, Kaelin C. Young, Stuart M. Phillips, Andreas N. Kavazis and Michael D. Roberts*

2575 The maturation of regional sweating patterns from childhood to young adulthood in females
*Hannah Blount, Nuno Koch Esteves, Jade Ward, Grant H Simmons, Peter R Worsley and Davide Filingeri*

### MUSCLE

2588 Neuromuscular fatigue and loss of torque complexity during severe-intensity exercise: The role of muscle size
*Rubens Correa Junior, Renan Vieira Barreto, Gabriel Fontanetti, Leonardo Coelho Rabello de Lima, Benedito Sérgio Denadai and Camila Coelho Greco*

2599 Bed rest decreases resting skeletal muscle $O_2$ uptake and resting energy expenditure in young and elderly subjects
*Giovanni Baldassarre, Simone Porcelli, Lucrezia Zuccarelli, Lorenza Brocca, Noemi Arboit, Laura Gobbo, Michele Lacerenza, Alessio Marciano, Chiara Motto, Giulia Sanguin, Mladen Gasparini, Boštjan Šimunič, Rado Pišot, Marco Narici and Bruno Grassi*

2613 Metabolomic responses are more sensitive in muscle than serum following 28 days of arduous exercise with erythropoietin administration
*Devin J. Drummer, Emily E. Howard, Julie L. McNiff, Marques A. Wilson, Christopher T. Carrigan, Nancy E. Murphy, Jess A. Gwin, David E. Barney Jr, Benjamin J. Ryan, James P. McClung, Stefan M. Pasiakos and Lee M. Margolis*

2627 Lipid emulsion modulates myogenic and collagen-related gene expression in skeletal muscle of preterm fetal sheep
*Xinrui Li, Sarah M Alaniz, Samantha Louey, Jeanene Marie Deavila, Sonnet S. Jonker and Min Du*

### NEUROSCIENCE

2637 Sex-based differences in corticospinal excitability and inhibition
*Alicia M. Kells, Alexandra N. Pauhl and Anita D. Christie*

2645 Adaptive mediolateral control during split-belt walking: Energetics of interlimb coordination and enhanced savings following acute intermittent hypoxia
*Norah M. Nyangau, Alysha T. Bogard, Aviva K. Pollet and Andrew Q. Tan*

2665 Menstrual cycle effects on sex differences in motor control during dynamic balance perturbations
*Samuli Nevanperä, Ritva S. Mikkonen, Simon Walker and Jarmo M. Piirainen*

### RESPIRATORY

2682 Influence of low-dose dopamine on exercise in fibrosing interstitial lung disease
*Charlotte Chen, Jui-Lin Fan, Michael Plunkett, Thalia Babbage, John Kolbe, Julian F. R. Paton and James P. Fisher*

2696 Respiratory and non-respiratory airflow characteristics across ingestive and non-ingestive swallowing tasks
*Elizabeth Cross, Esther Guiu Hernandez and Phoebe Macrae*

2713 Moderate maternal nutrient restriction alters type II alveolar epithelial cell density in the non-human primate fetal lung
*Mitchell C. Lock, Hillary F. Huber, Cun Li, Sandra Orgeig, Peter W. Nathanielsz and Janna L. Morrison*

### LETTER TO THE EDITOR

2729 On the statistical interpretation of ultrasound-based models predicting skeletal muscle fat infiltration
*Saul Martin-Rodriguez*

Editor's Pick

Clustering dendrograms of metabolites co-expressing in module assignments for serum (top) and skeletal muscle (bottom). Coloured bars indicate module assignments for clustered metabolites. See Drummer et al. Pages 2613–2626.
